# Correspondence

**Published:** 1862-07

**Authors:** W. H. Butler

**Affiliations:** Washington


### CORRESPONDENCE.

WASHINGTON, June 20, 1862.

Washington now may be called a City of Hospitals. There are no fewer than twelve regularly named and occupied in the limits of the city, and the churches and other buildings lately taken will swell the number to nearly thirty—one at Georgetown and three or four buildings used for hospital purposes at Alexandria. There is also one hospital at Falls Church and at Fairfax C. H. In these are collected a great variety of diseases, gun-shot wounds, &c. It has been my effort to see as many of the interesting cases as possible, and with that view have visited several of the hospitals. While at the "Circle Hospital," Drs. Connover and Robbins related a case of puerperal peritonitis, in which they were associated. The woman was confined Sunday: Monday, she got up and did some washing, and in the afternoon a disturbance occurred in the house which greatly excited her. Tuesday she felt pain in the abdomen and sent for medical help, but got none till evening, when Drs. C. and R. visited her. Then she was in great agony, with thighs flexed, unable to bear the slightest pressure over the region of the abdomen, and had been vomiting; pulse 195. No doubt existed in the minds of the medical men as to the cause being inflammatory. The patient was given two grains of opium every few minutes until in two hours she had taken nineteen grains. The medical gentlemen remained that time, the pulse having come down to about one hundred, and the woman being calm. No unpleasant consequences followed the administration of this large quantity of opium. Of the purity of the article there can be no question, as it was of a lot inspected by the U. S. Inspector, and in daily use at the hospital.

This was the woman's seventh confinement, a stout washing-woman, wife of a private soldier. The case is interesting as showing how large a quantity of this narcotic may be borne in this grave disease, as well as a remarkable recovery under the circumstances.

Dr. Bowen, of the Circle Hospital, also showed me three worms, (*ascarides lumbricoides,*) two of which were found in making a post mortem on a typhoid fever patient, lying in the jejunum. This person was of strong constitution, and about thirty years of age. In another case occurring in the hospital, that of a boy sixteen years of age, one of the round worms crawled out of his mouth, he being at the time in a comatose condition, and very low, but has entirely recovered.

In speaking of this subject in the society of a party of medical gentlemen, and making the enquiry whether any had noticed the same fact. Dr. Bigelow of Grand Rapids, Mich., mentioned the case of a strong, hearty girl, of fourteen years of age, that had a round worm crawl from the nose during the height of a severe attack of typhoid fever, occurring last winter, who ultimately recovered.

I am not familiar with any notice having before this been taken of an association of worms with this type of fever; they may be entirely accidental; at any rate it is worth enquiring of the profession at large if such circumstances have before been noticed.

Many, or perhaps I should say several, secondary operations have been done in the hospitals in the city since the late battles, rendered necessary by the hopelessness of recovery without. Amputation at the shoulder was performed three days ago, the man doing well, and the very pleasant weather for the past week has been of great benefit to all our sick and wounded. Among the many cases related to me, that of a private, wounded at Fair Oaks, through the superior maxilla, is of interest. In this case the common carotid was ligated on the field, the man making a good recovery. W. H. BUTLER, M. D.

